# Editorial: Viral infection, tumor development and host immune response

**DOI:** 10.3389/fcimb.2023.1177032

**Published:** 2023-03-10

**Authors:** Xin Li, Liwei An, Yucui Jin, Jun Gui, Weitao Lin, Zhenke Wen, Muzaffer Ahmad Kassab, Jie Chen

**Affiliations:** ^1^ Chinese Academy of Sciences (CAS) Key Laboratory of Pathogenic Microbiology and Immunology, Institute of Microbiology, Chinese Academy of Sciences (CAS), Beijing, China; ^2^ Department of Stomatology, Shanghai Tenth People’s Hospital, Tongji University School of Medicine, Shanghai, China; ^3^ Department of Biochemistry and Molecular Biology, Tongji University School of Medicine, Shanghai, China; ^4^ Department of Medical Genetics, Nanjing Medical University, Nanjing, China; ^5^ State Key Laboratory of Oncogenes and Related Genes, Med-X Clinical Stem Cell Research Center, Renji Hospital, School of Medicine, Shanghai Jiao Tong University, Shanghai, China; ^6^ Harry Perkins Institute of Medical Research, QEII Medical Centre and Centre for Medical Research, The University of Western Australia, Nedlands, WA, Australia; ^7^ Centre for Precision Health, Edith Cowan University, Joondalup, WA, Australia; ^8^ Jiangsu Key Laboratory of Infection and Immunity, Institutes of Biology and Medical Sciences, Soochow University, Suzhou, China; ^9^ Department of Cancer Biology, University of Pennsylvania, Philadelphia, PA, United States

**Keywords:** viral infection, inflammation, cancer, immune response, precise prevention and precise treatment

Cancer is a devastating disease that remains a leading cause of death worldwide. According to recent statistics, cancer was responsible for 10 million deaths in 2020 alone (Ferlay et al., 2021). Of these cases, viral infections associated cancers have been found to contribute to a significant proportion, with chronic infections such as hepatitis B and C viruses (HBV and HCV), human papillomavirus (HPV), Epstein-Barr virus (EBV), and Human immunodeficiency virus (HIV) being particularly noteworthy. Inflammation and immune response are also closely linked to tumorigenesis, making it crucial to understand how viral infections and host immune response contribute to cancer development (Anand et al., 2008; White et al., 2014). To this end, a special Research Topic has been prepared to advance our knowledge in this field.

Mounting studies demonstrated chronic HBV infection might be associated not only with an increased risk of hepatocellular carcinoma but also with the extrahepatic malignancies, such as gastric cancer (GC). Several epidemiological studies have found that the risk of GC was increased by chronic HBV infection. However, a quantitative result addressing the association between HBV infection and GC development as well as mutation profiles of HBV-infected patients with GC is poorly understood. In this Research Topic, Li et al. revealed the relationship and the mechanism between chronic HBV infection and GC through meta-analysis and serum epidemiological analysis. HBV infection was associated with a significantly higher risk of GC when compared with the healthy controls without HBV infection. Besides, the authors identified unique mutation profiles in HBV infected GC samples, such as mutated KMT2B, FGF12, and TUBB2B, which may have implications in GC development. The plausible mechanisms underlying the association between HBV infection and the development of GC might be correlated to HBV infection-induced persistent inflammation, immune dysfunction, and cirrhosis. In addition, integration of the viral genome has been considered as one of the mechanisms for malignant transformation in gastric epithelial cells. Overall, the authors’ research added new insights for investigating the mechanism of HBV-induced GC.

Cervical cancer (CC) is one of the most prevalent cancers among women worldwide, with the majority of cases resulting from HPV infection. The transport and processing (TAP) of foreign antigens, carried out by TAP proteins, play a crucial role in the immune system by presenting pathogenic peptides to CD8+ cytotoxic T cells (CTLs) and natural killer (NK) cells and that helps clear viral infections. Single-nucleotide polymorphisms (SNPs) in TAP genes can lead to evasion of the immune response. However, the relationship between HPV infection and CC, in association with the SNPs of TAP genes, remains an enigma. To delineate the relationship, Medeiros et al. conducted a comprehensive investigation of SNPs, gene expression, and protein levels of TAPs from hundreds of patient samples to demonstrate that SNPs in TAP genes make HPV-infected patients susceptible to the development of high-grade cervical lesions. The study showed that SNPs in TAP genes increased the risk of chromosomal alterations in cervical cells and precancerous lesions, and HPV-infected women had higher levels of TAP2 in mRNA and protein. These findings provide profound insight into the mechanism of CC caused by HPV and that could help in futuristic drug developments against this cancer.

Altered energy metabolism is an essential hallmark of cancer. However, the role of altered energy metabolism in host cells in modulating latent and lytic EBV infection in nasopharyngeal carcinoma (NPC) cells remains unclear. Latent EBV infection and expression of latent EBV genes have been shown to drive alterations in cellular metabolism to modulate the malignant phenotypes of NPC cells. Here, Yang et al. review the impact of genetic alterations in NPC on cellular metabolism and their impact on the establishment of latent infection and lytic reactivation of EBV infection in NPC cells. They also comprehensively update the roles of EBV-encoded genes in driving glucose metabolism and their contribution to NPC pathogenesis. Furthermore, they provided a perspective on the interplay between EBV infection and altered host metabolic pathways in NPC growth and malignant properties, which may offer novel and effective therapeutic vulnerabilities in the treatment of NPC and other EBV-associated malignancies.

AIDS-related lymphoma (ARL) is a major cause of mortality in individuals with HIV infection. Patients with ARL are typically diagnosed with diffuse large B-cell lymphoma (DLBCL) or Burkitt lymphoma (BL), and these diseases tend to be more aggressive in HIV-positive patients than those without HIV. Unfortunately, prognostic assessment for patients with ARL remains poor. To address this issue, Chen et al. performed a retrospective multicenter cohort study of 138 primary ARL patients over an 8-year period. They identified age, extranodal sites, bulky mass, CD4 T-cell counts, CD4/CD8 ratio, and hypoalbuminemia as significant prognostic factors for overall survival (OS). Furthermore, the study found that the CD4/CD8 ratio was a powerful independent prognostic parameter in ARL patients. When the CD4/CD8 ratio was integrated into the International Prognostic Index (IPI), the composite HIV-IPI score provided a more accurate prognostic assessment, with potential implications for risk stratification and guiding therapeutic decisions for ARL patients.

In conclusion, this special Research Topic sheds light on the mechanisms by which viral infections drive tumor development and provides insights into how this knowledge can guide the prognostic assessments and therapeutic decisions. We express our gratitude to all authors for their contributions to this Research Topic. Their studies have significantly advanced our understanding of the role of viral infections in cancer and paved the way for further research in these fields. However, given the widespread involvement of viruses in different types of cancer, further investigations are needed, and we hope that future efforts in these Research Topics will continue to expand our knowledge in these areas.

## Author contributions

XL, LA, YJ, JG, WL, ZW, MK, and JC wrote the Editorial. All authors contributed to the article and approved the submitted version.
